# Evaluation of Sensory and Motor Function in Spinal and Bulbar Muscular Atrophy Using Quiet Stance and Reactive Postural Control

**DOI:** 10.3390/neurolint17060079

**Published:** 2025-05-22

**Authors:** Joseph A. Shrader, Ashwini Sansare, Allison C. Niemic, Rafael Jiménez-Silva, Joshua G. Woolstenhulme, Galen O. Joe, Uma Jacobs, Angela Kokkinis, Kenneth Fischbeck, Chris Grunseich, Cris Zampieri

**Affiliations:** 1 Rehabilitation Medicine Department, Clinical Center, National Institutes of Health, Bethesda, MD 20892, USA; 2 Neurogenetics Branch, National Institute of Neurological Disorders and Stroke, National Institutes of Health, Bethesda, MD 20892, USAchristopher.grunseich@nih.gov (C.G.)

**Keywords:** Spinal Bulbar Muscular Atrophy, Kennedy’s Disease, balance, posturography, strength, automatic postural reflexes, sensory postural control

## Abstract

Introduction: Spinal and bulbar muscular atrophy (SBMA) is an X-linked neuromuscular disorder characterized by progressive muscle weakness, along with muscle cramps, tremors, and sensory neuropathy. Previous research has shown that patients with SBMA have difficulty with dynamic balance and sensory postural control during quiet stance. There have been no reports on automatic postural reactions in SBMA. Objectives: In this study, we aimed (1) to augment previous findings of sensory postural control, (2) to investigate automatic postural reactions in SBMA, and (3) to explore the relationship between strength and balance. Design: A cross-sectional design was used for the analysis. Participants: The participants were fifty male individuals with a confirmed diagnosis of SBMA. Outcome Measures: Balance testing included the NeuroCom modified Clinical Test of Sensory Interaction on Balance (mCTSIB), which measures sway velocity during quiet stance, and the NeuroCom Motor Control Test (MCT), which measures the latency and strength of postural reactions following sudden perturbations. Strength testing included maximal voluntary isometric contractions measured via fixed-frame dynamometry. Results: Forty-seven out of fifty participants were able to complete the mCTSIB test, but only thirty-eight completed the MCT test. Patients who were unable to complete the MCT were significantly weaker in all lower extremity muscles compared to those who were able to complete testing. Compared to normative data, participants showed significantly higher sway velocity during quiet stance across all conditions of the mCTSIB, except when standing on foam with eyes open. They also exhibited significantly slower postural reactions in response to sudden shifts of the force plate on the MCT. Plantarflexor weakness was significantly correlated with poor postural control on the mCTSIB and MCT. Conclusions: This study confirms previously reported abnormalities of sensory postural control in SBMA and highlights patients’ heavy reliance on visual inputs for postural control. Additionally, this study shows that automatic postural corrections are slower than normal in SBMA and provides a unique approach for measuring the combined sensory and motor components of the disease. Both the sensory and automatic balance abnormalities were found to be associated with plantarflexor weakness and may contribute to a higher risk of falls under challenging situations. Therefore, addressing this weakness may be an important step toward fall prevention in this population.

## 1. Introduction

Spinal and bulbar muscular atrophy (SBMA), also known as Kennedy’s Disease (KD), is an X-linked neuromuscular disorder caused by the toxic effects of a mutation in the androgen receptor gene affecting both muscle and motor neuron cells [1]. The disease has an adult onset and causes slowly progressive weakness of the bulbar and extremity muscles. Sensory neuropathy has also been reported [2]. The damage to skeletal muscle can lead to muscle cramps, tremors, and decreased or absent deep tendon reflexes, and it will invariably have a negative impact on patients’ daily functioning and endurance [2]. 

Lower extremity weakness in patients with KD affects dynamic balance, as shown in a study involving 50 men who performed forward lunges, step quick turns, and stepping onto and over an obstacle [3]. This study found that (1) at least 35% of the cohort were too weak to perform the above-mentioned tasks safely, (2) those who were able to complete the tasks scored significantly worse on every balance measure compared to normative values, and (3) knee extensor and ankle plantarflexor weakness were related to their ability to perform the dynamic tasks. Impairments of static balance have also been reported in KD [4]. In a study involving seven men, Anagnostou et al. [4] detected excessive postural sway, especially when eyes were closed. Clinical exams confirmed that all participants had abnormal sensory (sural) nerve action potentials and five of seven participants had normal vestibular function, which prompted the authors to conclude that the postural instability seen was due to proprioceptive deficits. Given that this was a small group of participants, there is a need for research in larger groups to confirm these findings. Since balance is a complex construct, there are other aspects of postural control that have yet to be explored in SBMA, such as automatic reactions [5]. Automatic postural reactions are reflexes that allow an individual to promptly regain steadiness following a postural perturbation (slip or trip). These reactions are fundamental for safe ambulation and have been found to be impaired in patients with multiple sclerosis and traumatic brain injury, with the authors suggesting that delayed postural reactions could increase falls risk [6,7]. In the present study, we aim to measure sensory postural control in a larger group of participants with KD, investigate automatic postural reactions to unexpected perturbations, and explore associations between strength and balance.

## 2. Materials and Methods

### 2.1. Participants

Fifty males (mean age: 55.0 ± 9.0 years; age range: 29–74 years; disease duration: 15.6 years ± 9.2 years) included in this cross-sectional analysis were initially recruited for participation in a larger randomized controlled trial investigating the effects of functional exercise for individuals with SBMA (NCT01369901) [8]. Inclusion criteria required that participants were ambulatory, over 18 years of age, diagnosed with SBMA as confirmed by genetic testing, and scored between 14 and 41 on the Adult Myopathy Assessment Tool (AMAT) [9]. This tool includes 13 performance-based items that provide a total score ranging from 0–45 and includes subscales for muscle strength and endurance. The analysis only includes data from their baseline visit before the exercise regimen had been initiated. The trial protocol was approved by the NIH Institutional Review Board and informed consent was obtained from all the participants. Patients’ scores were compared to reference values provided by the manufacturer of the balance testing system (SMART Equitest^®^ System by NeuroCom (previously Natus Medical Inc., Middleton, WI, USA). 

### 2.2. Outcome Measures

Three outcome measures were investigated: the modified Clinical Test of Sensory Interaction on Balance (mCTSIB), the Motor Control Test (MCT), and maximal voluntary isometric contraction (MVIC). Posturography was utilized for mCTSIB and MCT, which were completed barefoot on a NeuroCom Balance SMART Equitest^®^ System (previously Natus Medical Inc., Middleton, WI, USA). Fixed-frame dynamometry was utilized for MVIC testing via Quantitative Muscle Assessment (Aeverl Medical, Gainesville, GA, USA). 

Sensory control of posture: The mCTSIB is a test of quiet stance which measures the use of sensory information (visual, vestibular, and somatosensory) for balance [10]. Participants stand quietly and completely still three times, for 10 s, under four different conditions: on a firm surface with eyes open, on a firm surface with eyes closed, on a foam surface with eyes open, and on a foam surface with eyes closed. The outcome measure of this test is the sway velocity of the center of gravity (degrees/second). Higher scores indicate worse balance. The average of three trials was analyzed. 

Automatic control of posture: The MCT assesses the automatic postural response of a subject to unexpected external perturbations. Participants stand on two movable force plates that generate sudden translations of small (0.5 inch), medium (1 inch), and large (1.5 inches) magnitudes in the forward and backward directions. We only analyzed large backward translations to mimic a forward stumble. Participants stand as steady as possible with one foot on each force plate and their weight evenly distributed. The first outcome measure of this test is the latency of their automatic response, defined as the time lapse, in milliseconds, between the onset of the force plate translation and the automatic active force response of the subject, measured individually for each lower limb [11]. Higher scores indicate slower responses. The second outcome measure was the strength of the automatic plantarflexion response (amplitude scaling), a unitless value indicating the strength of the participant’s response in relation to each perturbation [12]. Abnormally high amplitude scaling demonstrates an over-correction, and abnormally low demonstrates an under-correction or possible motor weakness. Three trials were averaged for each leg. Any loss of balance or compensatory strategy by the subject was noted and the trials were interrupted. Data processing involved two steps: (1) computer-assisted visual inspection of weight symmetry and (2) software algorithm confidence. Weight distributions were not adequate for four patients and their data were excluded from the analyses. The software algorithm assigns confidence values of 0–4, with 0 representing the lowest confidence in the latency measurement and 4 representing the highest confidence. As recommended by the NeuroCom manufacturer (previously Natus Medical Inc., Middleton, WI, USA), we examined latency tracings for any trial with confidence values less than 2 to see if the program correctly determined when the motor response began. Nine patients had confidence value scores less than 2, with two of those being incorrectly marked by the program and manually corrected. The other seven patients were correctly marked.

Control data for Neurocom balance measures were obtained from the manufacturer and were reported to include individuals with no current or past diagnosis of injury affecting balance, no use of medications affecting the central nervous system or known to affect balance or coordination, no symptoms of dizziness or lightheadedness, no diagnosis of vestibular or neurological disorders, no psychological disorders, no history of two or more unexplained falls within the past 6 months, and normal vision, with or without glasses.

Strength: The muscle groups included in this analysis were hip extensors, knee extensors, ankle dorsiflexors, and ankle plantarflexors, measured bilaterally. Participants were instructed to exert their maximum force for two trials lasting 5 s each, and the average of the two trials was analyzed. Strength was recorded in kilograms, and percent of predicted strength was calculated according to previously published equations specific to gender, age, height, weight, muscle group, and side of the body [13]. Fixed-frame MVIC of ankle plantarflexion does not have a reliable normative equation; therefore, the percent of predicted strength was calculated by comparing strength to control ankle plantarflexion data previously measured in our laboratory with our equipment (10 healthy men, mean age 51 years, mean body mass index (BMI) 27.0).

### 2.3. Statistical Analysis

Shapiro–Wilk tests were performed to check all data for normality. Data were dichotomized into those who were able (participants who completed all three trials of a test without loss of balance) and those who were not able (participants who required reaching for upper extremity support, therapist assistance, or took a corrective step to prevent a fall) to complete all trials. mCTSIB and MCT measures were compared between patients who were able to complete each test and the normative database with an independent samples *t*-test, and their respective effect sizes were calculated with Cohen’s *d*. The following interpretation criteria were used for effect sizes: small (0.20), moderate (0.50), and large (0.80) [14]. Individual counts of abnormal values (more than two standard deviations relative to the normative database) were obtained for each test in the group of participants who were able to complete the tests. To determine the role of strength on balance results, MVIC values for lower extremity muscles were correlated with mCTSIB and MCT measures, among those able to complete the tests, with a Pearson correlation corrected for the age of each participant. Additionally, total lower extremity MVIC was compared between participants able to complete the MCT and those unable to do so with an independent samples *t*-test. The level of significance was kept at 0.05. Analyses were performed with R software (R Foundation for Statistical Computing, version 3.6.0, Vienna, Austria).

## 3. Results

Our cohort included 50 male subjects with a mean age of 55 ± 9 years and an average disease duration of 15.6 ± 9.2 years. Mean composite lower extremity strength was 55 ± 20 percent of predicted. Their mean functional performance level was moderate, with an AMAT mean score of 29 ± 6.7 out of a possible 45 points (higher scores indicate higher function). All 50 patients completed the first two conditions of the mCTSIB (firm surface eyes open and firm surface eyes closed), and 47 completed all four conditions. Three participants did not complete the full mCTSIB due to loss of balance while standing on the foam. Only 38 patients were able to complete the MCT. Six had a near fall during testing, four did not meet the required weight symmetry criteria, and two were deemed unsafe to try by the therapist. There was a significant difference in lower extremity strength between those able and unable to complete the MCT. The unable group was significantly weaker in all right-sided lower extremity muscles as well as the bilateral composite lower extremity muscles compared to the able group, with a moderate effect size for hip extension and large effect sizes in all other cases (Table 1). There was no significant difference in age (*p* = 0.23) or disease duration (*p* = 0.09) between the groups.

mCTSIB and MCT results in comparison to normative values for those able to complete each respective test are presented in Table 2. Participants were separated into age groups that matched the age groups of the normative dataset. The patient group displayed significantly higher sway velocities on three out of four conditions of the mCTSIB, regardless of age. Effect sizes were particularly large for all eyes-closed conditions. The MCT test results show that patients employed significantly longer latencies (reaction times after perturbations) compared to controls regardless of age (*p* < 0.001 for right and left latencies in both age groups). Effect sizes for latency differences were very large (*d* = 1.34–1.87). There were no group differences for the strength of plantarflexion response (*p* = 0.057–0.231). 

**Figure 1:**
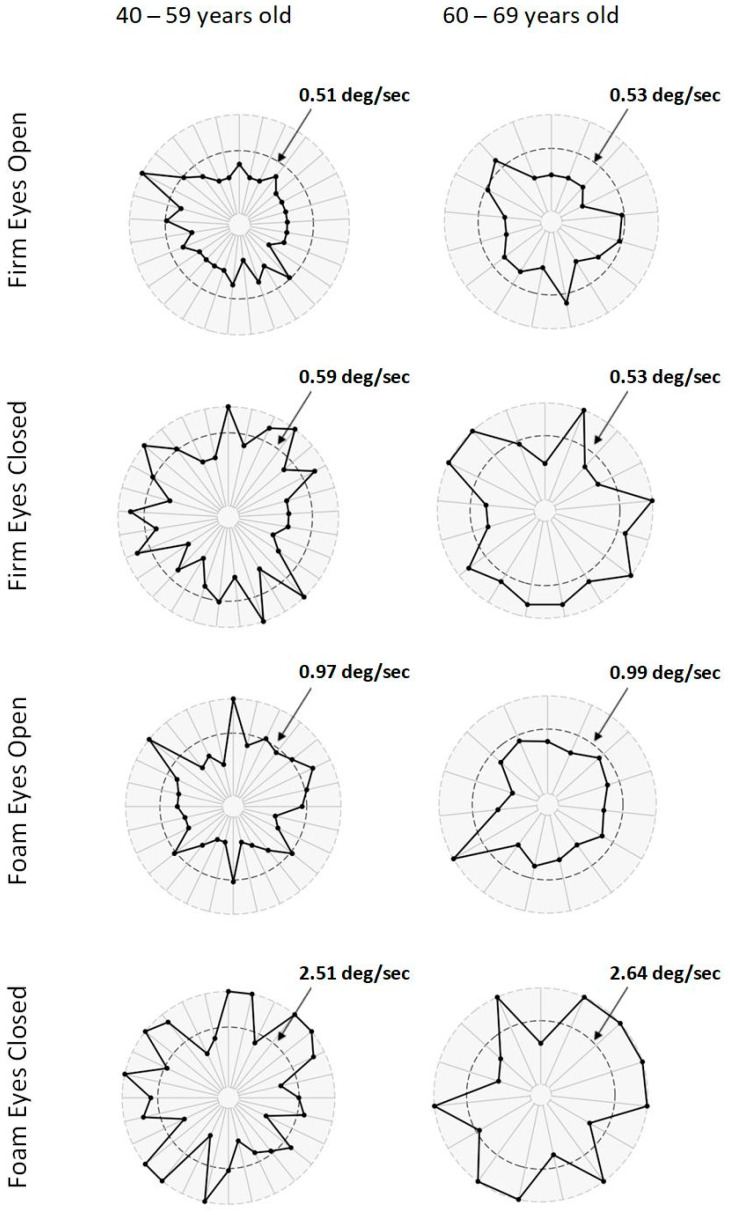
Individual patient results on the modified Clinical Test of Sensory Interaction on Balance (mCTSIB). Notes: Each row is a condition of the test, and each column is an age group. Each dot represents a participant’s center of gravity sway velocity. The further from the center of the graph, the higher, and worse, the result. The inner dashed circle delimits the normal cut point based on the normative mean ± two standard deviations; values are presented by condition and age group. Dots that fall outside the inner dashed circle are considered abnormal. Note that more patients scored abnormal on eyes-closed conditions.

An in-depth analysis exploring individual mCTSIB results is presented in Figure 1 to identify abnormal results (more than two standard deviations from the normative mean). Importantly, during all eyes-open conditions, most SBMA patients had sway velocity within normal ranges. In contrast, during eyes-closed conditions, most patients had abnormal sway, independent of the surface (firm or foam) or age group (40–59 years or 60–69 years).

**Figure 2:**
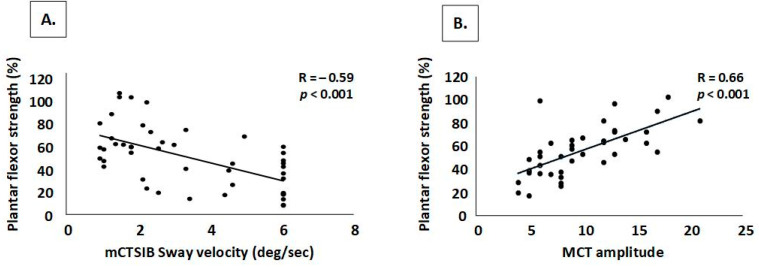
Correlation plots showing associations between strength and balance as measured by the mCTSIB (**A**) and MCT (**B**). Note: Plot (**A**) displays the correlation between bilateral plantarflexor percent of predicted strength and mCTSIB sway velocity measured during the foam eyes-closed condition. Plot (**B**) displays the correlation between the left plantarflexor percent of predicted strength and the left amplitude response measured during the MCT.

Correlations between strength and balance are shown in Figure 2. As plantarflexor strength decreased, postural sway on the mCTSIB increased. Additionally, weaker plantarflexors were associated with weaker automatic plantarflexion response on the MCT (Figure 2B). Not shown in the figure, the right-side plantarflexor association with MCT was nearly identical to that of the left (R = 0.58, *p* < 0.001) and composite lower extremity strength (R = 0.57, *p* < 0.001).

## 4. Discussion

This is the first investigation into reactive balance in people with SBMA. Sensory control of balance has been explored in a small group of men with SBMA [4]. The present study cohort had moderate to severe SBMA as indicated by their relatively low functional performance on AMAT testing (29/45), MVIC lower extremity composite strength values of only 55% of predicted, and disease duration of 15.5 years.

Despite these challenges, subjects remained ambulatory, with or without an assistive device, and sensory control of balance (mCTSIB) had a completion rate of 94%. Assessment of automatic postural responses (MCT) was more difficult for people with SBMA, with a completion rate of only 76%. However, both tests had a low floor effect compared with dynamic balance tests attempted by the same cohort (step-quick-turn, step-up-and-over, and forward lunge; 52–65% completion rates) [3]. We suggest that the mCTSIB and MCT have good clinical utility for this population.

### 4.1. Heavy Reliance on Visual Input with Sensory Control of Balance Testing

Participants in this study performed significantly worse than control subjects in both eyes-closed conditions of the mCTSIB with large effect sizes, irrespective of age. However, they did not perform worse than control subjects when standing on foam with eyes open, possibly indicating compensatory use of visual input to stabilize sway velocity. This is not unexpected, as previous research has shown that visual input reliance increases, and proprioceptive input reliance decreases, when healthy individuals move from standing on a stable to an unstable surface [15]. Further, we examined individual data (Figure 1) to assure that outliers were not affecting group data and confirmed more participants exhibited abnormal sway velocity under eyes-closed conditions compared to eyes-open conditions, irrespective of age and surface. Our findings augment previous results by Anagnostou et al., who reported significant abnormal sway compared to controls for the eyes-closed conditions [4]. The authors suggest proprioceptive deficits were responsible for their findings, having generally ruled out vestibular impairments and ruled in sensory neuron action potential deficits. However, we are not as confident in ruling out vestibular deficits in our cohort because subjects performed worse in both foam/eyes-closed and firm/eyes-closed conditions when each was normalized to their baseline (firm eyes-open condition). Unfortunately, we did not have access to sural nerve action potentials or diagnostic vestibular testing to better identify which system was more responsible for increased sway velocity. Our findings lead us to conclude that (1) visual inputs appear to rescue impaired sway and (2) both proprioceptive and vestibular systems seem to be impaired. Additionally, it is important to consider that mCTSIB testing assumes normal strength [10]. As shown above, we quantified relatively severe lower extremity muscle weakness in our cohort. Therefore, muscle weakness confounds the impact that both vestibular and proprioceptive inputs have on postural control. Finally, we note that despite considerable lower extremity muscle weakness, men with SBMA used visual inputs to demonstrate normal sway velocities when a certain level of strength was available. Previous research has shown that weakness at the ankle negatively impacts one’s ability to effectively use the ankle strategy to maintain postural control during tests exploring sensory feedback on balance [16].

Practical things to consider are obtaining/maintaining regular vision checkups and pre-planning for instances when visual deficits are likely during upright mobility tasks (e.g., when showering or walking with poor lighting).

### 4.2. Strength and Sensory Deficits Impact Automatic Postural Reactions

Strength was a possible determinant of our patients’ ability to complete the MCT. Those able to complete the MCT test were significantly stronger in composite lower extremity strength, at 53% of predicted versus only 40% for those unable to complete the test, whereas disease duration and age were not different. This finding may suggest that a strength threshold of approximately ≤40% could interfere with automatic postural responses in large backward floor translations in SBMA. Given the propensity toward tripping forward in SBMA, posturography, when available, may provide a safe test to simulate an unexpected slip or a trip [17]. 

Additionally, among those patients who could complete the MCT, we found that their automatic postural reactions were significantly delayed compared with control data (NeuroCom, manufacturer), with large effect sizes for both age groups. These findings are similar to those reported by Inglis et al. [18] in patients with somatosensory problems due to peripheral neuropathy, with a comparable age range to our cohort. These data are also consistent with previous studies showing similar latencies of this magnitude in multiple sclerosis [6] and traumatic brain injury [7]. 

Cameron et al. [6] noted patients with MS compensated for slow latencies by increasing the magnitude of their ankle plantarflexion strength responses. Our cohort exhibited slow latencies, and our younger participants showed a trend of utilizing this compensation by increasing their strength responses compared with control data (*p* = 0.057, with a medium effect size). We are unsure why our older group did not utilize this strategy. Nevertheless, we found that the plantarflexor MVIC of the entire cohort correlated with the strength of the automatic ankle plantarflexion response.

The clinical relevance of these findings may include the following: SBMA patients who are unable to complete the MCT test may have trouble recovering from an unexpected forward trip. Based on our findings in the unable group, when MVIC ankle plantarflexion strength was below 44% of predicted (mean ± SD), forward trip recovery was compromised. When MCT testing reveals large delays (latencies) in responding to unexpected forward trip perturbations (large backward translations), increased ankle plantarflexion MVIC could help patients compensate by generating a stronger strength response (amplitude response) as seen in some of our SBMA participants. Moreover, safely learning and practicing how to make a recovery step with clinical supervision may help patients with SBMA prevent a fall. 

### 4.3. Limitations and Future Directions

Nerve conduction tests were not included in the present study; therefore, it was not possible to measure the degree to which decreased sensory nerve action potentials contributed to the findings. However, previous studies have shown diminished sensory nerve action potentials [19] as well as loss of dorsal root ganglion cells seen at autopsy [20] in individuals with SBMA. More research is needed to clarify the confounding influence of muscle weakness in postural control. We suggest that future studies include objective MVIC testing of key lower extremity muscles along with tests of sensory neuron action potentials, vestibular function, and more specific balance tests, i.e., the sensory organization test and limits of stability. 

Additionally, while we would have preferred to use an age-matched control group, the design of the original study was to determine the effects of an exercise program rather than to explore balance deficits. For this reason, we were limited to the usage of manufacturer-derived normative data, which might have affected the accuracy of the balance measures given the wide age range for the control group. 

## 5. Conclusions

This study provides evidence that people with SBMA who have considerable strength and dynamic balance abnormalities (3) can largely complete objective static balance tests for the detection of impairments related to automatic postural responses and the use of sensory information for balance control. The data reveal significant delays in reactive postural control and impairments in using both vestibular and proprioceptive information to adequately maintain safe control of standing balance (sway velocity). Visual inputs appear to offer important advantages to improve balance in people with SBMA, and we suggest patients receive education about these findings. More research is needed to explore interventions to help people with SBMA maintain or improve lower extremity muscle strength and safely train to improve both static and dynamic balance.

## Figures and Tables

**Table 1:** Comparison of strength levels between patients able to perform the MCT versus those unable.

MVIC	Mean ± SD		*p* Value	Cohen’s *d*	ES
Muscle Group Tested	Able	Unable			
	*N* = 38	*N* = 12			
Knee extensors	42.3 ± 22.2	20.1 ± 10.5	0.002	1.10	Large
Ankle dorsiflexors	45.4 ± 18.3	30.2 ± 14.6	0.012	0.87	Large
Ankle plantarflexors	59.3 ± 22.1	25.8 ± 18.5	<0.001	1.57	Large
Hip Extensors	102.6 ± 38.4	77.3 ± 23.5	0.045	0.71	Moderate
Lower extremity composite	62.0 ± 19.4	37.4 ± 13.6	<0.001	1.35	Large

Notes: MVIC = maximum voluntary isometric contraction, ES = effect size. The unit of measure for MVIC is the percent of predicted strength, accounting for gender, age, height, weight, muscle group, and side of the body [12].

**Table 2:** Balance test performance of patients versus controls.

Test (Unit of Measure)	Age (Years)	Condition	Mean ± SD		*p* Value	Cohen’s *d*	ES
			Patient	Control			
mCTSIB	40–59		*N* = 29	*N* = 29			
(degrees/second)		Firm Eyes Open	0.36 ± 0.12	0.27 ± 0.12	**0.003**	0.72	Medium
		Firm Eyes Closed	0.78 ± 0.99	0.33 ± 0.13	**0.020**	0.73	Medium
		Foam Eyes Open	1.05 ± 1.20	0.63 ± 0.17	0.076	0.57	Medium
		Foam Eyes Closed	3.40 ± 1.74	1.61 ± 0.45	**0.000**	1.60	Large
	60–69		*N* = 17	*N* = 26			
(degrees/second)		Firm Eyes Open	0.38 ± 0.12	0.28 ± 0.12	**0.009**	0.86	Large
		Firm Eyes Closed	0.68 ± 0.30	0.31 ± 0.11	**0.000**	1.80	Large
		Foam Eyes Open	0.83 ± 0.45	0.69 ± 0.15	0.252	0.48	Small
		Foam Eyes Closed	4.11 ± 1.94	1.60 ± 0.52	**0.000**	2.03	Large
MCT	20–59		*N* = 25	*N* = 29			
(milliseconds)		Latency Left	143.6 ± 11.9	117.0 ± 19.8	**0.000**	1.60	Large
		Latency Right	140.0 ± 13.5	117.0 ± 19.8	**0.000**	1.34	Large
(unitless)		Strength Left	10.92 ± 4.43	8.60 ± 4.30	0.057	0.53	Medium
		Strength Right	9.24 ± 3.53	8.60 ± 4.30	0.550	0.16	Small
	60–69		*N* = 11	*N* = 54			
(milliseconds)		Latency Left	150.0 ± 14.8	124.0 ± 15.6	**0.000**	1.68	Large
		Latency Right	152.7 ± 14.2	124.0 ± 15.6	**0.000**	1.87	Large
(unitless)		Strength Left	8.27 ± 4.03	9.90 ± 3.40	0.231	0.47	Small
		Strength Right	7.73 ± 4.15	9.90 ± 3.40	0.127	0.62	Medium

Notes: Performance of patients who were able to complete mCTSIB and MCT compared to a normative database of control subjects. The unit of measure for mCTSIB is sway velocity of the center of gravity, measured in degrees/second. MCT measures are latency of response (milliseconds), either right or left, and strength = amplitude of the strength response (unitless), either right or left. Significant *p* values are bolded. Two 70-year-old participants were excluded from the analysis due to a lack of normative data for comparison.

## Data Availability

The de-identified data used to support the findings of this study are available from Christopher Grunseich, christopher.grunseich@cc.nih.gov, for researchers who meet the criteria to access the data.

## References

[1] Kennedy W.R., Alter M., Sung J.H. (1968). Progressive proximal spinal and bulbar muscular atrophy of late onset. A sex-linked recessive trait. Neurology.

[2] Rhodes L.E., Freeman B.K., Auh S., Kokkinis A.D., La Pean A., Chen C., Lehky T.J., Shrader J.A., Levy E.W., Harris-Love M. (2009). Clinical features of spinal and bulbar muscular atrophy. Brain.

[3] Shrader J.A., Sansare A., Shieh V., Woolstenhulme J.G., Rekant J., Jiménez-Silva R., Joe G.O., Kokkinis A., Fischbeck K.H., Grunseich C. (2021). Dynamic Balance in Spinal and Bulbar Muscular Atrophy: Relationship between Strength and Performance of Forward Lunge, Step Up and Over, and Step Quick Turn. Rehabil. Res. Pract..

[4] Anagnostou E., Zachou A., Breza M., Kladi A., Karadima G., Koutsis G. (2019). Disentangling balance impairments in spinal and bulbar muscular atrophy. Neurosci. Lett..

[5] Mancini M., Horak F.B. (2010). The relevance of clinical balance assessment tools to differentiate balance deficits. Eur. J. Phys. Rehabil. Med..

[6] Cameron M.H., Horak F.B., Herndon R.R., Bourdette D. (2008). Imbalance in multiple sclerosis: A result of slowed spinal somatosensory conduction. Somatosens. Mot. Res..

[7] Newton R.A. (1995). Balance abilities in individuals with moderate and severe traumatic brain injury. Brain Inj..

[8] Shrader J.A., Kats I., Kokkinis A., Zampieri C., Levy E., Joe G.O., Woolstenhulme J.G., Drinkard B.E., Smith M.R., Ching W. (2015). A randomized controlled trial of exercise in spinal and bulbar muscular atrophy. Ann. Clin. Transl. Neurol..

[9] Harris-Love M.O., Fernandez-Rhodes L., Joe G., Shrader J.A., Kokkinis A., La Pean Kirschner A., Auh S., Chen C., Li L., Levy E. (2014). Assessing function and endurance in adults with spinal and bulbar muscular atrophy: Validity of the adult myopathy assessment tool. Rehabil. Res. Pr..

[10] Nashner L.M., Peters J.F. (1990). Dynamic posturography in the diagnosis and management of dizziness and balance disorders. Neurol. Clin..

[11] Vanicek N., King S.A., Gohil R., Chetter I.C., Coughlin P.A. (2013). Computerized dynamic posturography for postural control assessment in patients with intermittent claudication. J. Vis. Exp..

[12] The National Isometric Muscle Strength (NIMS) Database Consortium (1996). Muscular weakness assessment: Use of normal isometric strength data. Arch. Phys. Med. Rehabil..

[13] Portney L. (2015). Foundations of Clinical Research: Applications to Practice.

[14] Peterka R., Loughlin P. (2004). Dynamic regulation of sensoriomotor integration in human postural control. J. Neurophysiol..

[15] Jacobson G.P., Newman C.W., Kartush J.M. (1997). Handbook of Balance Function Testing.

[16] Robinovitch S.N., Heller B., Lui A., Cortez J. (2002). Effect of strength and speed of torque development on balance recovery with the ankle strategy. J. Neurophysiol..

[17] Lockhart T.E., Woldstad J.C., Smith J.L., Ramsey J.D. (2002). Effects of age-related sensory degradation on perception of floor slipperiness and associated slip parameters. Saf. Sci..

[18] Inglis J.T., Horak F.B., Shupert C.L., Jones-Rycewicz C. (1994). The importance of somatosensory information in triggering and scaling automatic postural responses in humans. Exp. Brain Res..

[19] Pelosi L., Ghosh A., Leadbetter R., Lance S., Rodrigues M., Roxburgh R. (2022). Nerve ultrasound detects abnormally small nerves in patients with spinal and bulbar muscular atrophy. Muscle Nerve.

[20] Li M., Sobue G., Doyu M., Mukai E., Hashizume Y., Mitsuma T. (1995). Primary sensory neurons in X-linked recessive bulbospinal neuropathy: Histopathology and androgen receptor gene expression. Muscle Nerve.

